# Epigallocatechin gallate (EGCG) inhibits African swine fever virus replication by targeting key viral proteins and regulating host lipid metabolism

**DOI:** 10.1080/22221751.2026.2731508

**Published:** 2026-09-07

**Authors:** Xinglin He, Hong Li, Ruiqi Yang, Pengfei Li, Guiqian Wang, Hua Cao, Yumei Sun, Mengjia Zhang, Yongtao Li, Kaizhi Shi, Anan Jongkaewwattana, Shengnan Ruan, Wentao Li

**Affiliations:** a College of Veterinary Medicine, Hainan Research Institute, National Key Laboratory of Agricultural Microbiology, Huazhong Agricultural University, Wuhan, People’s Republic of China; b Key Laboratory of Preventive Veterinary Medicine in Hubei Province, The Cooperative Innovation Center for Sustainable Pig Production, Wuhan, People’s Republic of China; c Hubei Jiangxia Laboratory, Wuhan, People’s Republic of China; d College of Veterinary Medicine, Henan Agricultural University, Zhengzhou, People’s Republic of China; e Institute of Animal Husbandry and Veterinary Medicine, Guizhou Academy of Agricultural Sciences, Guiyang, People’s Republic of China; f Virology and Cell Technology Research Team, National Center for Genetic Engineering and Biotechnology (BIOTEC), National Science and Technology Development Agency (NSTDA), Pathum Thani, Thailand

**Keywords:** Epigallocatechin gallate (EGCG), African swine fever virus (ASFV), antiviral activity, viral protein targeting, lipid metabolism

## Abstract

African swine fever (ASF) is a highly pathogenic swine infectious disease caused by African swine fever virus (ASFV), with a mortality rate approaching 100% in domestic pigs and causing severe economic losses to the global pig industry. Despite the recent approval of live-attenuated ASF vaccines in limited regions such as Vietnam, universally safe, globally authorized commercial vaccines and effective antiviral therapeutics remain unavailable, creating an urgent demand for innovative anti-ASFV intervention strategies. In this study, epigallocatechin gallate (EGCG) was identified to exert prominent inhibitory effects on ASFV proliferation *in vitro*. Viral life cycle assays indicated that EGCG mainly targets the internalization and post-entry genome replication stages. Molecular docking combined with bio-layer interferometry (BLI) confirmed high-affinity direct binding between EGCG and two indispensable ASFV proteins, p72 and p1192R. Furthermore, EGCG dose-dependently activates the AMP-activated protein kinase (AMPK) signalling pathway, downregulates lipid synthesis-related genes, and reverses ASFV-induced lipid droplet accumulation and abnormal increases in total cholesterol (TC), triglycerides (TG), and free fatty acids (FFAs), thereby disrupting the lipid metabolic microenvironment required for viral replication. Collectively, EGCG suppresses ASFV replication through dual mechanisms: targeting key viral proteins and regulating host lipid metabolism. This study highlights EGCG as a promising anti-ASFV candidate and offers a novel theoretical basis for the development of anti-ASFV agents.

## GRAPHICAL ABSTRACT

**Figure:**
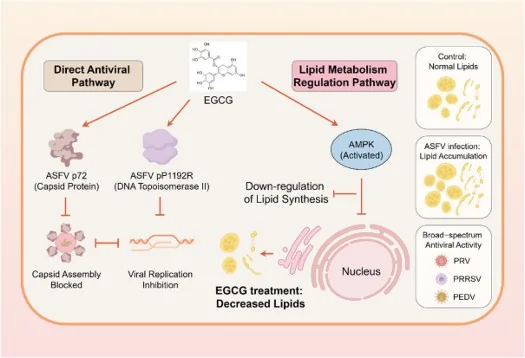
EGCG suppresses ASFV replication through dual mechanisms: targeting viral key proteins and regulating host lipid metabolism.

## Introduction

ASF is an acute, febrile, and highly contagious disease of pigs caused by ASFV infection. Its typical clinical features include high fever, systemic hemorrhagic lesions, and multiple organ failure, with a mortality rate of up to 100% in domestic pigs [1,2]. Since its first report in Kenya in 1921, ASF has spread to many countries and regions around the world, inflicting devastating blows to the global swine industry, causing immeasurable economic losses, and seriously threatening food safety and social stability [3–7].

ASFV is a large, structurally complex double-stranded DNA virus and the only member of the family Asfarviridae. Its genome is approximately 170–193 kb in length, encoding more than 150 proteins, endowing the virus with strong immune evasion and environmental adaptation capabilities [8–10]. The complexity of the virus also poses great challenges to vaccine development. Although candidate vaccines such as attenuated live vaccines and subunit vaccines have shown certain protective effects in recent years, key issues such as their safety, genetic stability, and cross-protective ability have not been fully resolved [11–16]. Recently, live attenuated vaccines have been licensed for commercial use in Vietnam; however, their global application remains limited due to ongoing surveillance of long-term safety and efficacy [11,17]. To date, universally approved commercial ASF vaccines or specific therapeutic drugs are still lacking worldwide [14]. Current epidemic control mainly relies on strict biosecurity measures, early diagnosis, and culling of infected animals [18–20]. This grim situation highlights the necessity of developing novel anti-ASFV strategies, especially exploring efficient and safe antiviral drugs or lead compounds [21].

Natural products have become an important resource library for antiviral drug development due to their rich biological activities, good safety, and multi-target action characteristics [22–26]. In this study, we conducted a molecular docking-based virtual screening campaign, evaluating natural compounds against the available 3D crystal structures of ASFV replication and structural proteins. We identified that EGCG, the major bioactive polyphenol derived from green tea, possesses potent natural anti-ASFV activity. EGCG has been confirmed to exhibit various biological activities, including antibacterial, antiviral, anti-inflammatory, and metabolic regulation [27–31]. In antiviral research, the mechanisms of EGCG have been validated against multiple viruses [29,32]. For example, EGCG can exert antiviral effects by targeting key molecules such as influenza virus hemagglutinin protein and hepatitis B virus core antigen, or by regulating host cell autophagy and inflammatory signalling pathways [33–35]. Studies have found that ASFV replication depends on host cell lipid metabolism, and viral infection can induce the accumulation of lipid droplets in host cells, providing energy and structural materials for viral envelope assembly and genome replication [36,37]. As a core regulator of cellular energy metabolism, AMPK is closely associated with ASFV-induced lipid metabolism disorders. However, the mechanism by which EGCG inhibits ASFV replication has not been reported to date.

This study aims to investigate the anti-ASFV activity of EGCG and its underlying mechanisms. The results confirmed that EGCG can effectively inhibit ASFV replication *in vitro*, and its antiviral effect is achieved through a dual mechanism. On the one hand, molecular docking and functional validation demonstrated that EGCG exhibits strong interactions with the key ASFV proteins p72 and p1192R, forming stable complexes that interfere with the viral replication cycle by directly targeting these viral proteins. On the other hand, EGCG activates the AMPK signalling pathway, downregulates the expression of key host lipid synthesis genes, and disrupts the lipid metabolic microenvironment essential for ASFV replication, thereby suppressing viral proliferation [36]. This study systematically clarifies the molecular mechanism underlying the anti-ASFV activity of EGCG, providing a solid theoretical basis and promising candidate molecule for the development of novel anti-ASFV strategies targeting host lipid metabolism or key viral proteins.

## Materials and methods

### Cells and viruses

LLC-PK1, PK-15, Marc-145, and WSL cells were cultured in Dulbecco’s modified Eagle’s medium (DMEM; Gibco, USA) supplemented with 10% fetal bovine serum (FBS; ExCell Bio, China), 100 U/mL penicillin, and 100 μg/mL streptomycin. Porcine alveolar macrophages (PAMs) were isolated from 28-day-old specific pathogen-free (SPF) pigs (Approval Code: HZAUSW-2026-0067) and cultured in RPMI 1640 medium (Gibco, USA) containing 10% FBS, 100 U/mL penicillin, and 100 μg/mL streptomycin under identical incubation conditions. For viral strains, the genotype II strain ASFV-0428C and recombinant ASFV-GFP were constructed in our laboratory as previously described [38,39]. Recombinant genotype I and genotype II ASFV strains ASFV-HN10005 (ASFV-HN) were obtained from the National African Swine Fever Regional Laboratory at South China Agricultural University [40]. PRRSV-GFP, PRV-GFP, and PEDV-GFP were preserved in our laboratory [41–43]. All experiments involving live ASFV, including virus propagation, infection, and sample processing, were performed in a biosafety level-3 (BSL-3) laboratory in accordance with national biosafety regulations. All experimental waste was subjected to high-pressure steam sterilization at 121 °C for 30 min or chemically inactivated to ensure complete pathogen elimination prior to disposal.

### Chemicals and reagents

Epigallocatechin gallate (EGCG, ≥98% purity) was acquired from Sigma-Aldrich (St. Louis, USA). Stock solution at 10 mM was prepared in DMSO and stored at −80 °C in the absence of light. Dorsomorphin, a well-characterized AMPK inhibitor, was purchased from MedChemExpress (MCE, NJ, USA). Serial working concentrations of EGCG and dorsomorphin were freshly diluted with cell culture medium before use, with the final DMSO proportion maintained below 0.1% (v/v) to exclude solvent-induced cellular toxicity. All custom oligonucleotide primers and fluorescent probes for quantitative real-time PCR were chemically synthesized by Sangon Biotech (Shanghai, China). Fluorescent secondary antibodies including Cy3®-conjugated goat anti-mouse IgG H&L and Alexa Fluor® 488-conjugated goat anti-mouse IgG H&L were purchased from Abcam (Shanghai, China) and AntGene (Wuhan, China), respectively. Additional secondary antibodies (goat anti-mouse IgG and goat anti-pig IgG), as well as antibodies targeting FLAG-tag, Strep-tag, and the internal loading control β-actin, were sourced from ABclonal Biotechnology (Wuhan, China). Primary antibodies against total AMPK and phosphorylated AMPK (p-AMPK) were procured from Cell Signaling Technology (Danvers, MA, USA). The mouse monoclonal antibody against ASFV p72 and ASFV-positive porcine convalescent serum were independently prepared and preserved in our laboratory. Commercial biochemical assay kits for the quantitative detection of triglycerides (TG), total cholesterol (TC), and free fatty acids (FFA) were supplied by Applygen Technologies Inc. (Beijing, China). The fluorescent dye BODIPY 493/503 (Invitrogen, USA) was utilized for specific staining and visualization of intracellular lipid droplets.

### Cytotoxicity assay

The cytotoxicity of the compounds in cells was detected using Cell Counting Kit-8 (CCK-8) assays to determine cell viability. Briefly, cell suspensions (100 µL/well) were seeded into 96-well plates and incubated at 37 °C in a humidified atmosphere containing 5% CO_2_. Cells were treated with various concentrations of compounds and incubated for an additional 48 h. Following treatment, 10 µL of CCK-8 reagent was added to each well containing 100 µL of fresh DMEM, followed by incubation at 37 °C for 2 h. The absorbance at 450 nm was measured using a microplate reader to assess cell viability. Cytotoxicity analysis was performed according to the manufacturer’s protocol.

### Virus titration

Viral titres were determined using the Reed-Muench method. Briefly, ASFV-infected cells were subjected to three freeze–thaw cycles at −80 °C to release intracellular virions, and the supernatant was collected. The viral suspension was then serially 10-fold diluted in cell culture medium and added to 96-well cell monolayers (100 µL per well) with eight replicates per dilution. After 3-5 days of incubation at 37 °C in a CO_2_ incubator, cytopathic effects (CPE) or characteristic fluorescence were observed and recorded. The TCID_50_ was subsequently calculated according to the classical Reed-Muench method.

### Real-time qPCR

ASFV genomic DNA was extracted using the FastPure Viral DNA/RNA Mini Kit (Vazyme, Nanjing, China) according to the manufacturer’s instructions. Viral genome copy numbers were quantified by qPCR using a CFX Connect Real-Time PCR Detection System (Bio-Rad). The primers and probe sequences were as follows: forward primer, 5′-CTGCTCATGGTATCAATCTTATCGA-3′; reverse primer, 5′-GATACCACAAGATCRGCCGT-3′; probe, 5′-FAM-CCACGGGAGGAATACCAACCCAGTG-TAMRA-3′.

For cellular gene expression analysis, total RNA was extracted from cells using TRIzol Reagent (TaKaRa) according to the manufacturer’s instructions. The extracted RNA was reverse-transcribed into cDNA using the PrimeScript RT reagent kit (Vazyme, Nanjing, China). Quantitative real-time PCR (qRT-PCR) was performed in triplicate using SYBR Premix Ex Taq (Vazyme, Nanjing, China) on a CFX Connect Real-Time PCR Detection System. The expression levels of target genes were normalized to that of GAPDH, and the relative quantification was calculated by the 2^−ΔΔCt^ method. The specific primer sequences used for evaluating host lipid metabolism-related gene expression are comprehensively listed in Supplementary Table S4.

To characterize the transcriptional kinetics of ASFV early and late genes during infection, specific primer pairs were designed against the early gene *CP204L* (encoding the p30 protein) and the late gene *B646L* (encoding the major capsid protein p72). The corresponding primer sequences were listed as follows: *CP204L* forward, 5′- CGGTAGAATTGTTACGAC-3′; *CP204L* reverse, 5′- TTCTTGAGCCTGATGTTC-3′; *B646L* forward, 5′-CCACGTAATCCGTGTCCCAA-3′; *B646L* reverse, 5′- GATGATCCGGGTGCGATGAT-3′.

### Immunofluorescence assay

ASFV-infected cells were washed with PBS and fixed with 3.7% paraformaldehyde (PFA) for 10 min at room temperature (RT), followed by permeabilization with 1% Triton X-100 in PBS for 10 min. After blocking with 5% bovine serum albumin (BSA) in PBS for 45 min at RT, cells were incubated with anti-p72 mouse monoclonal antibody (1:2000) at 37 °C for 1 h, then stained with Alexa Fluor® 488-conjugated or Cy3-conjugated goat anti-mouse IgG secondary antibody (1:1000). Nuclei were counterstained with DAPI for 10 min at RT before fluorescence imaging using a laser scanning confocal microscope.

### Quantitative image analysis

Fluorescent imaging was performed using a confocal laser scanning microscope. To ensure unbiased and reproducible quantification, at least three random microscopic fields were captured for each experimental group, covering approximately 10 individual cells per biological replicate. Quantitative analysis of intracellular lipid droplets (LDs) was implemented using ImageJ software (NIH, USA). Briefly, background fluorescence was subtracted uniformly from all images, and a consistent binary threshold was applied to segment LD-positive signals. The Area Fraction parameter was subsequently determined to calculate the proportion of LD fluorescent area relative to the total cellular area within each field of view, which was defined as the relative LD occupancy (% Area).

### Western blotting analysis

To evaluate the host AMPK signalling pathway, ASFV-infected or mock-infected cells were lysed on ice using RIPA lysis buffer supplemented with protease and phosphatase inhibitor cocktails. Total protein concentrations were determined using a BCA Protein Assay Kit. Equal amounts of protein were separated by SDS-PAGE and transferred onto PVDF membranes. After blocking, the membranes were incubated overnight at 4°C with specific primary antibodies, including anti-AMPK (1:2000), anti-phospho-AMPK (1:2000), and anti-β-actin (1:2000) as an internal loading control. Following incubation with HRP-conjugated secondary antibodies (1:5000), protein bands were visualized using an enhanced chemiluminescence (ECL) detection system.

### Time-of-addition assay

Cells were seeded in 24-well plates and grown to confluence for the time-of-addition assay. EGCG was added at different time points relative to ASFV infection, as outlined in the schematic workflow, and cells were harvested at 24 hpi for viral genomic DNA quantification via qPCR, as described above. For stage-specific validation assays, viral adsorption and virucidal effect were evaluated by pre-incubating ASFV with EGCG at 37 °C for 1 or 2 h, adding the mixture to cells for 2 h of adsorption at 4 °C, incubating with fresh medium for 24 h; viral internalization was evaluated by incubating cells with ASFV at 4 °C for 2 h to allow binding, incubating at 37 °C with or without EGCG for 1, 2, or 4 h for internalization, treating with 0.25% trypsin for 5 min to remove surface-bound viruses, and incubating with fresh medium for 24 h; viral replication was validated by infecting cells with ASFV at 37 °C for 1 h, incubating with fresh medium for 2 or 4 h to establish replication, treating with EGCG, culturing for an additional 20 or 18 h respectively, with viral genomic DNA quantified via qPCR.

### Molecular docking analysis

The three-dimensional (3D) crystal structures of ASFV p72 and p1192R were utilized for molecular docking analysis. The chemical structure of EGCG was retrieved from the PubChem repository, and geometric optimization was performed in ChemDraw; subsequent structural preprocessing and visualization were completed in PyMOL. Molecular docking simulations were implemented using AutoDock Vina. The binding pocket regions of p72 and p1192R were delineated centred on their core catalytic/oligomerization functional domains to constrain the docking grid.

### Expression, purification, and validation of recombinant proteins

The recombinant ASFV p72 and p1192R proteins were expressed using a mammalian HEK293F cell expression system and subsequently purified via affinity chromatography. Crucially, to ensure the proper folding and authentic assembly of the major capsid protein p72, the viral chaperone protein B602L (Supplementary Figure S2A) was co-transfected and co-expressed during the production process. To strictly verify that the purified recombinant proteins maintained their native-like structural conformations and antigenic epitopes, their reactogenicity was evaluated using ASFV-positive swine serum. Immunoblot analysis confirmed that both the purified p72 and p1192R proteins were strongly and specifically recognized by the infected pig sera. This robust antigenic recognition demonstrates the preservation of their native functional folding prior to subsequent interaction assays.

### Bio-layer interferometry (BLI) analysis

BLI analysis was performed on an Octet RED96 system (ForteBio, USA). Streptavidin (SA) biosensors were first equilibrated in running buffer (PBS with 0.05% Tween-20, 0.1% BSA, pH 7.4) for 180 s to stabilize the baseline. Biotinylated recombinant p72 or p1192R protein (20 μg/mL) was loaded onto the SA biosensors for 420 s to achieve effective immobilization. EGCG was serially diluted to 0, 6.25, 12.5, 25, 50, 100, 200, 400 μM in the same running buffer, and the protein-immobilized biosensors were incubated with each EGCG dilution at 30 μL/min for 120 s, followed by a 600 s dissociation phase in running buffer without EGCG. All BLI sensorgrams were analyzed using Octet Data Analysis Software 12.0, and the dissociation constant (Kd) was calculated by fitting the binding curves to a 1:1 Langmuir binding model.

### CRISPR/Cas9-mediated viral gene disruption assay

CRISPR/Cas9 technology was employed to target and disrupt the ASFV *B646L* (encoding the p72 protein) and *P1192R* genes during viral infection in LLC-PK1-Cas9 cells. Specific single-guide RNAs (sgRNAs) targeting these viral genes were designed using the CRISPR Design Tool (http://crispr.mit.edu/). The sgRNA sequences were as follows: p72-sgRNA1: 5′- ACATTATATATGGCATCAGGAGG-3′, p72-sgRNA2: 5′- TTCCCTAGACGAATATAGTTCGG-3′; P1192R-sgRNA1: 5′-GGGCTTTGCGTCGGCCTTAATGG-3′, P1192R-sgRNA2: 5′- CTATAGCCTGCACCCCCTGCAGG-3′. The synthesized sgRNAs were cloned into a lentiviral expression vector. To generate lentiviral particles, the recombinant plasmids were co-transfected with matching packaging plasmids into HEK-293T cells. Supernatants containing lentiviruses were harvested to transduce LLC-PK1-Cas9 cells, and stable sgRNA-expressing cell lines were subsequently established via puromycin selection as previously described [44]. For the viral disruption validation, these stable cell lines were infected with ASFV-GFP at an MOI of 0.5. And viral replication was qualitatively assessed by observing the number of GFP-positive cells using a fluorescence microscope, and progeny viral titres in the cell supernatants were quantified via TCID50 assay to rigorously evaluate the essential roles of the p72 and p1192R proteins in ASFV replication.

### Determination of intracellular TC, TG and FFA

The intracellular levels of total cholesterol (TC), triglycerides (TG), and free fatty acids (FFA) were quantified using commercial colorimetric assay kits strictly according to the manufacturer’s instructions. The protein concentration of cell lysates was determined using a BCA protein assay kit to normalize lipid levels. Results were expressed as mmol/g protein for TC and TG, and μmol/g protein for FFA.

### BODIPY staining for lipid droplets

Cells were washed with PBS and fixed with 3.7% PFA for 15 min at room temperature (RT), followed by incubation with BODIPY 493/503 (excitation 493 nm / emission 503 nm) for 30 min in the dark to stain intracellular lipid droplets. Nuclei were subsequently counterstained with DAPI for 10 min at RT. Following final washes, fluorescence imaging was performed using a laser scanning confocal microscope. Quantitative image analysis of the lipid droplets was conducted using ImageJ software as described above.

### Statistical analysis

All experiments were performed independently at least three times, and data are presented as the mean ± standard deviation (SD). Statistical analyses were performed using GraphPad Prism software. Prior to parametric testing, data were evaluated for normal distribution and homogeneity of variances. Differences between two independent groups were analyzed using an unpaired two-tailed Student’s t-test. For comparisons among three or more groups with a single independent variable, a one-way analysis of variance (ANOVA) followed by Dunnett’s or Tukey’s post-hoc test was employed. For longitudinal data or experiments involving two independent variables, a two-way ANOVA followed by appropriate multiple comparison tests was utilized. Statistical significance was defined as *p < 0.05, **p < 0.01, and ***p < 0.001. Results with *p*≥0.05 were considered nonsignificant (ns).

## Results

### EGCG inhibits ASFV replication *in vitro*

**Figure 1.**
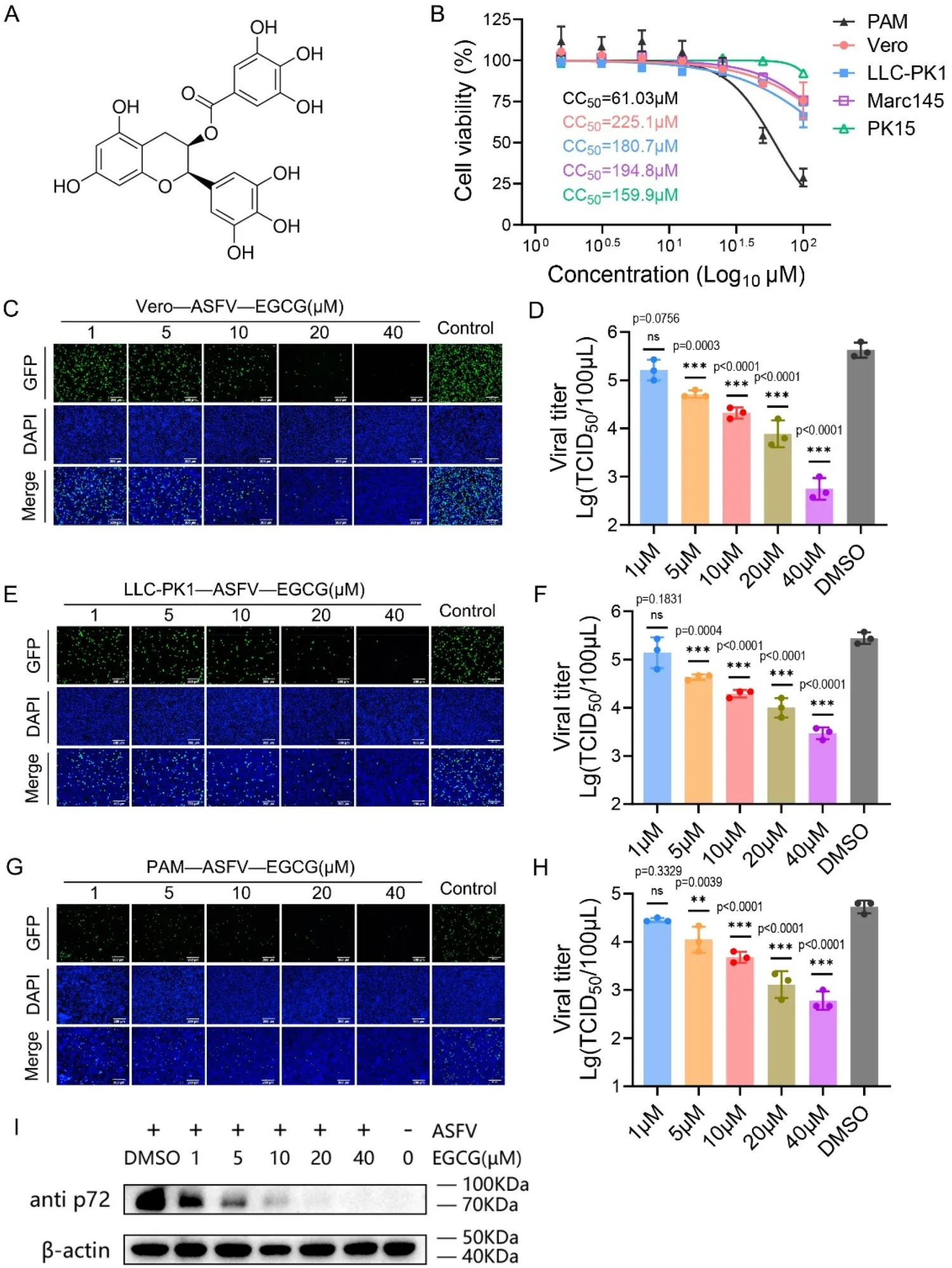
**EGCG inhibits ASFV replication *in vitro*.** (A) Chemical structure of epigallocatechin gallate (EGCG). (B) EGCG cytotoxicity across multiple cell lines. PAM, Vero, LLC-PK1, Marc-145, and PK-15 cells were incubated with gradient EGCG concentrations (0–100 μM) for 48 h, and cell viability was assessed by CCK-8 assay. (C, E, G) Fluorescence microscopy analysis of ASFV-GFP replication in Vero (C), LLC-PK1 (E), and PAM (G) cells infected with ASFV-GFP at an MOI of 0.5 and fixed and imaged at 48 hpi. GFP fluorescence indicates viral replication, and nuclei were counterstained with DAPI. Scale bars, 200 μm. (D, F, H) Viral titres in the supernatants of Vero (D), LLC-PK1 (F), and PAM (H) cells infected with ASFV at an MOI of 0.5 and harvested at 72 hpi, determined by TCID₅₀ assay. (I) Immunoblot analysis of ASFV p72 protein expression in Vero cells infected with ASFV and treated with indicated concentrations of EGCG or DMSO for 48 h. Data in (D, F, H) represent the mean ± SD of three independent biological replicates (n = 3). Statistical significance for panels was assessed by One-way ANOVA followed by Dunnett's multiple comparison test. ns, not significant; *p < 0.05, **p < 0.01, ***p < 0.001.

To accurately evaluate the inhibitory effect of EGCG on ASFV proliferation, recombinant ASFV-GFP was employed to assess viral replication. First, the cytotoxicity of EGCG on three commonly used ASFV-susceptible cell lines (Vero cells, LLC-PK1 cells, and PAMs) was assessed using the CCK-8 assay to determine its non-cytotoxic concentration range. The results demonstrated that cell viability remained above 90% in Vero and LLC-PK1 cells and over 80% in PAM cells at EGCG concentrations up to 40 μM (Figure 1(B)). To fully benchmark the safety and potency profiles of EGCG, the 50% cytotoxic concentration (CC_50_), the 50% inhibitory concentration (IC_50_), and the corresponding selectivity index (SI = CC_50_/IC_50_) were calculated (Supplementary Table S1). Notably, EGCG exhibited robust antiviral potency with IC_50_ values ranging from 5.2 to 8.3 μM, while maintaining a highly favourable safety profile with SI values ranging from 11.7 to 33.1, underscoring its viability as a safe antiviral candidate. Previous studies have reported that EGCG possesses low general cytotoxicity and does not actively induce non-specific cell cycle arrest or cell death under therapeutic thresholds, supporting its suitability for subsequent antiviral activity assays [45–47]. Next, we evaluated the inhibitory effect of different concentrations of EGCG on ASFV in Vero, LLC-PK1, and PAM cells. The results showed that EGCG inhibited ASFV proliferation in a dose-dependent manner. Fluorescence microscopy revealed that as the EGCG concentration increased, the GFP fluorescence signal in Vero (Figure 1(C)), LLC-PK1 (Figure 1(E)), and PAM cells (Figure 1(G)) significantly decreased and was nearly eliminated in the 40 μM treatment group. Viral titration further confirmed that EGCG treatment reduced progeny ASFV titres by 2–3 orders of magnitude in Vero cells (Figure 1(D)), LLC-PK1 cells (Figure 1(F)), and PAM cells (Figure 1(H)). Moreover, the expression of the ASFV core structural protein p72 was significantly suppressed in EGCG-treated cells (Figure 1(I)). Collectively, these results demonstrate that EGCG has a significant inhibitory effect on ASFV *in vitro* in a concentration-dependent manner.

### EGCG targets the post-entry replication stage of ASFV infection

**Figure 2.**
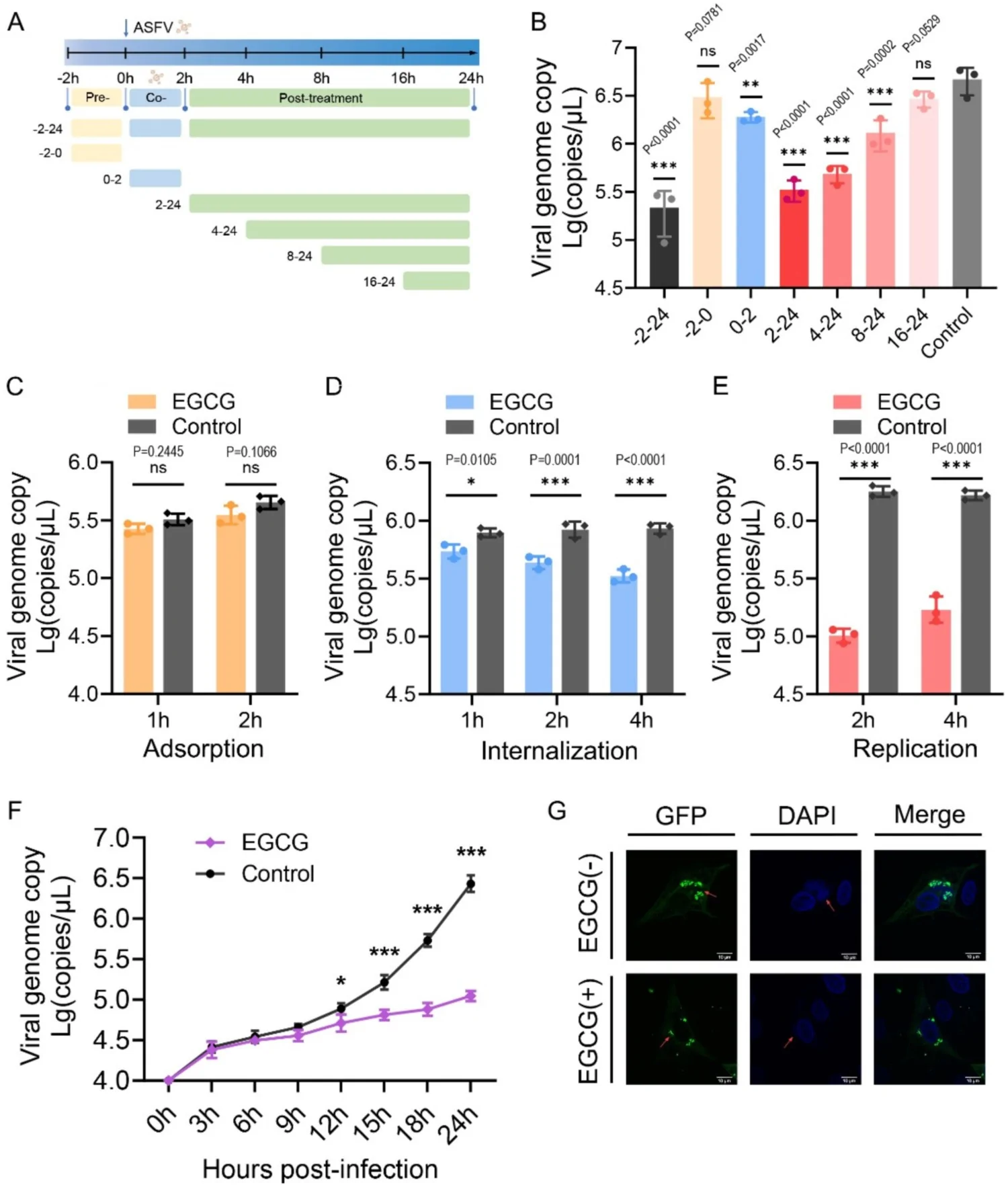
**EGCG targets the post-entry replication stage of ASFV infection.** (A) Schematic illustration of the time-of-addition experimental design. EGCG was administered at discrete time windows post-infection; DMSO served as vehicle control. Cells were collected at 24 hpi for qPCR quantification of intracellular viral genomic loads. (B) Viral genome copies quantified by qPCR at 24 hpi for the different treatment intervals illustrated in (A). Cells were infected with ASFV at an MOI of 0.5. (C, D, E) Stage-specific validation assays. Viral genome copies were measured during the viral adsorption (C), internalization (D), and replication (E) stages following treatment with EGCG (40 μM) or vehicle control. (F) Temporal profiles of viral genomic DNA accumulation. ASFV-infected cells (MOI = 0.5) were cultured with or without 40 μM EGCG, and viral genome copies were quantified at the indicated time points post-infection. (G) Confocal microscopy analysis of viral factory formation. Cells were infected with ASFV (MOI = 0.5) and treated with 40 μM EGCG. Red arrows mark characteristic cytoplasmic ASFV replication factories. Scale bars, 10 μm. Data are presented as the mean ± SD of three independent biological replicates (n = 3). Statistical significance for (B) was determined using One-way ANOVA followed by Dunnett’s multiple comparison test against the control group. Statistical significance for the time-course data in (C, D, E, F) was determined using Two-way ANOVA followed by Bonferroni's multiple comparison test to compare the EGCG-treated group with the control at each specific time point. ns, not significant; *p < 0.05, **p < 0.01, ***p < 0.001.

To clarify the mechanism by which EGCG inhibits ASFV proliferation, we investigated its effect on key stages of the ASFV life cycle. Time-of-addition assays were performed to identify the susceptible phases. EGCG was added at various time points (Figure 2(A)), and viral loads were determined at 24 h post-infection (hpi). The most potent inhibition was observed when EGCG was administered between 2 and 8 hpi, reducing viral genome copies by approximately 1 log unit (Figure 2(B)). Given that ASFV completes entry and internalization within approximately 2 hpi before initiating genome replication [48], we hypothesized that EGCG might interfere with both viral internalization and subsequent replication.

To further define the specific stages targeted by EGCG, a series of stage-specific assays were performed. First, ASFV was preincubated with EGCG for 1 or 2 h prior to cell inoculation to evaluate direct virucidal activity or effects on viral adsorption. The results showed that EGCG did not affect the viral adsorption phase (Figure 2(C)). For the internalization phase, cells were incubated with ASFV at 4 °C for 2 h to permit viral adsorption, followed by incubation at 37 °C in the presence or absence of EGCG for 1, 2, or 4 h. For the replication phase, cells were infected with ASFV at 37 °C for 1 h to enable adsorption and entry, followed by additional incubation for 2 or 4 h to establish viral replication before EGCG treatment. Viral genome copy numbers at each stage were quantified by qPCR. The results revealed that while ASFV internalization was mildly impaired (Figure 2(D)), a much more pronounced and significant inhibitory effect was observed during the replication stage (Figure 2(E)).

To further dissect the exact molecular stage of the replication cycle blocked by EGCG, we compared the transcript dynamics of the viral early gene (CP204L/p30) and late gene (B646L/p72) via RT-qPCR. As demonstrated in Supplementary Figure S1A, EGCG treatment exhibited negligible inhibitory effects on the transcription of the early gene CP204L during the initial stages of infection, indicating that EGCG does not interfere with the primary transcription machinery packaged within the incoming ASFV core particles. Conversely, EGCG treatment induced a catastrophic collapse in the mRNA levels of the late gene B646L (Figure S1B), alongside a massive restriction of viral genomic DNA replication (Figure 2(F)). Consistent with the profound blockade of DNA replication, confocal microscopy analysis visually confirmed that EGCG treatment thoroughly disrupted the formation of characteristic localized ASFV viral factories in the host cytoplasm (Figure 2(G)). Collectively, these phase-specific transcriptional and imaging data firmly establish that EGCG explicitly blocks the transition from early transcription to viral DNA genome replication and late morphogenesis.

### Molecular docking demonstrated robust binding affinity between EGCG and p72/p1192R proteins

**Figure 3.**
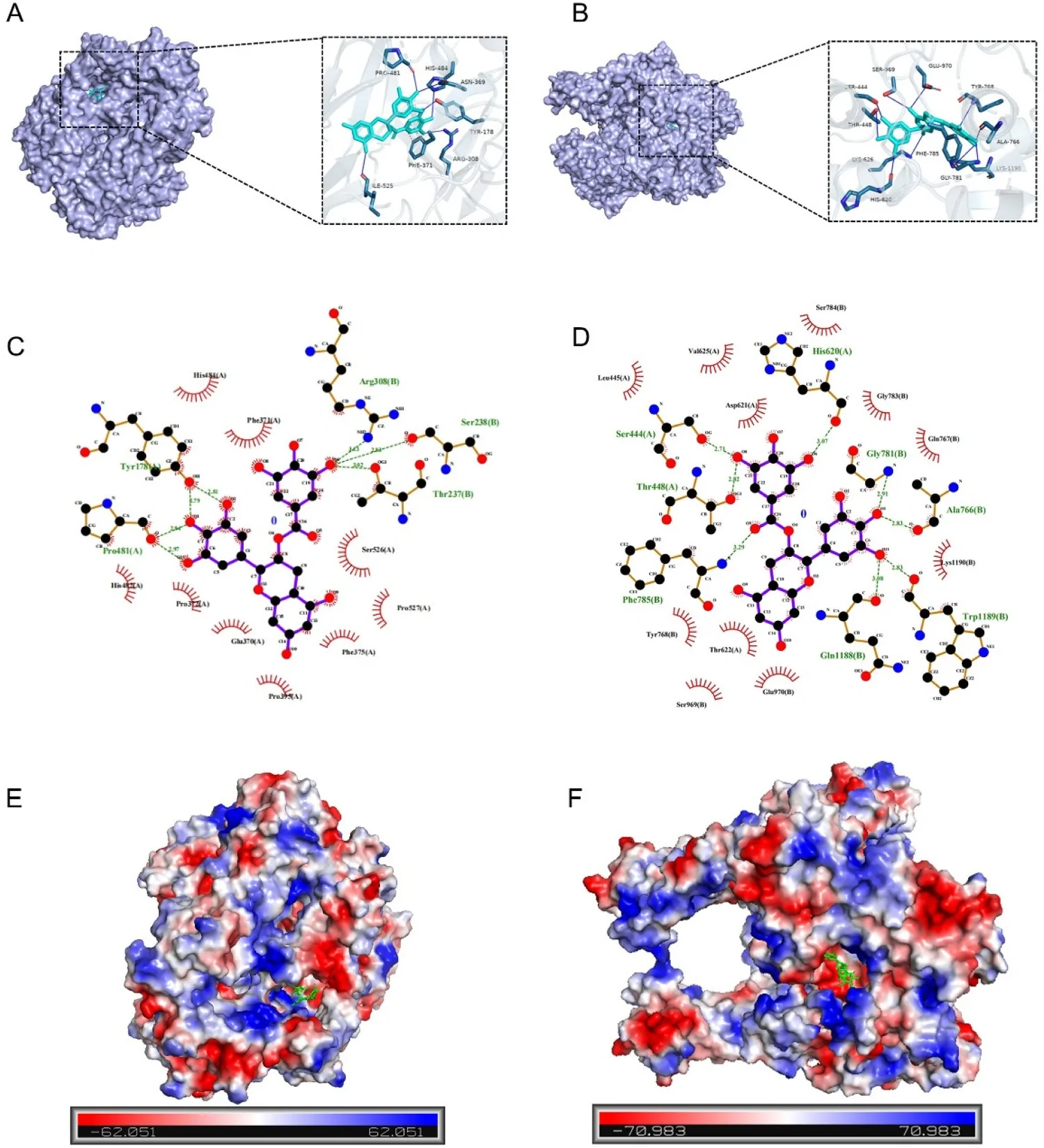
**Molecular docking and structural analysis of EGCG binding to ASFV p72 and p1192R proteins.** (A, B) 3D visualization of the optimal docking poses of EGCG bound to the p72 (A) and p1192R (B) proteins. The insets display zoomed-in views of the binding pockets, highlighting the interacting amino acid residues and EGCG. (C, D) 2D schematic representations of the detailed molecular interactions between EGCG and the surrounding residues of p72 (C) and p1192R (D). (E, F) Surface electrostatic potential maps of the p72 (E) and p1192R (F) proteins in complex with EGCG (shown in green). The molecular surface is coloured according to the electrostatic potential, ranging from negatively charged (red) to positively charged (blue) regions, with the colour scales provided at the bottom.

To determine whether EGCG directly interacts with ASFV proteins and uncover the molecular basis for its antiviral activity, we analyzed the binding potential of EGCG toward viral proteins using molecular docking. The results showed that EGCG exhibited strong binding potential with both ASFV p72 and p1192R proteins. As shown in Figure 3(A and B), molecular docking analysis revealed that EGCG adopted stable spatial conformations within the binding pockets of p72 and p1192R, with calculated optimal binding free energies of −9.3 kcal/mol and −10.0 kcal/mol, respectively. Detailed 2D interaction profiling (Figure 3(C and D)) revealed that the ligand formed highly efficient intermolecular interaction networks with key residues via robust hydrogen bonds and hydrophobic contacts. Furthermore, surface electrostatic potential maps (Figure 3(E and F)) demonstrated that the binding sites of both proteins displayed characteristic electrostatic distributions. Local negatively charged regions established favourable electrostatic complementarity with the polar moieties of EGCG, providing a structural explanation for the high-affinity binding from an electrostatic perspective. These results structurally validate the high binding affinity of EGCG toward ASFV p72 and p1192R proteins.

To evaluate the broad-spectrum antiviral potential of EGCG against diverse ASFV field isolates, we performed a comprehensive multiple sequence alignment of the p72 and p1192R target proteins. The analysis included representative highly pathogenic Genotype II strains (China/LN/2018/1, ASFV-SY18, Pig/HLJ/2018, and Georgia 2007/1), established Genotype I strains (BA71, L60, and OURT 88/3), as well as distinctive endemic isolates like ASFV-Ken.riel and ASFV/RWA/Musanze/2023. The alignment data revealed that the specific amino acid residues comprising the EGCG binding pockets are exceptionally conserved. For the p72 capsid protein, the critical EGCG-interacting residues (Tyr178, Arg308, Asn369, Phe371, Pro481, His484, and Ile525) exhibited 100% sequence identity across all analyzed genotypes. Similarly, the EGCG-anchoring residues within the p1192R topoisomerase II (including His620, Lys626, Tyr768, Gly781, Glu970, and Lys1190) demonstrated absolute conservation across the diverse viral panel. These alignment results are summarized in Supplementary Figures S3 and S4.

### EGCG interacts with p72 and p1192R proteins to impair viral replication

**Figure 4.**
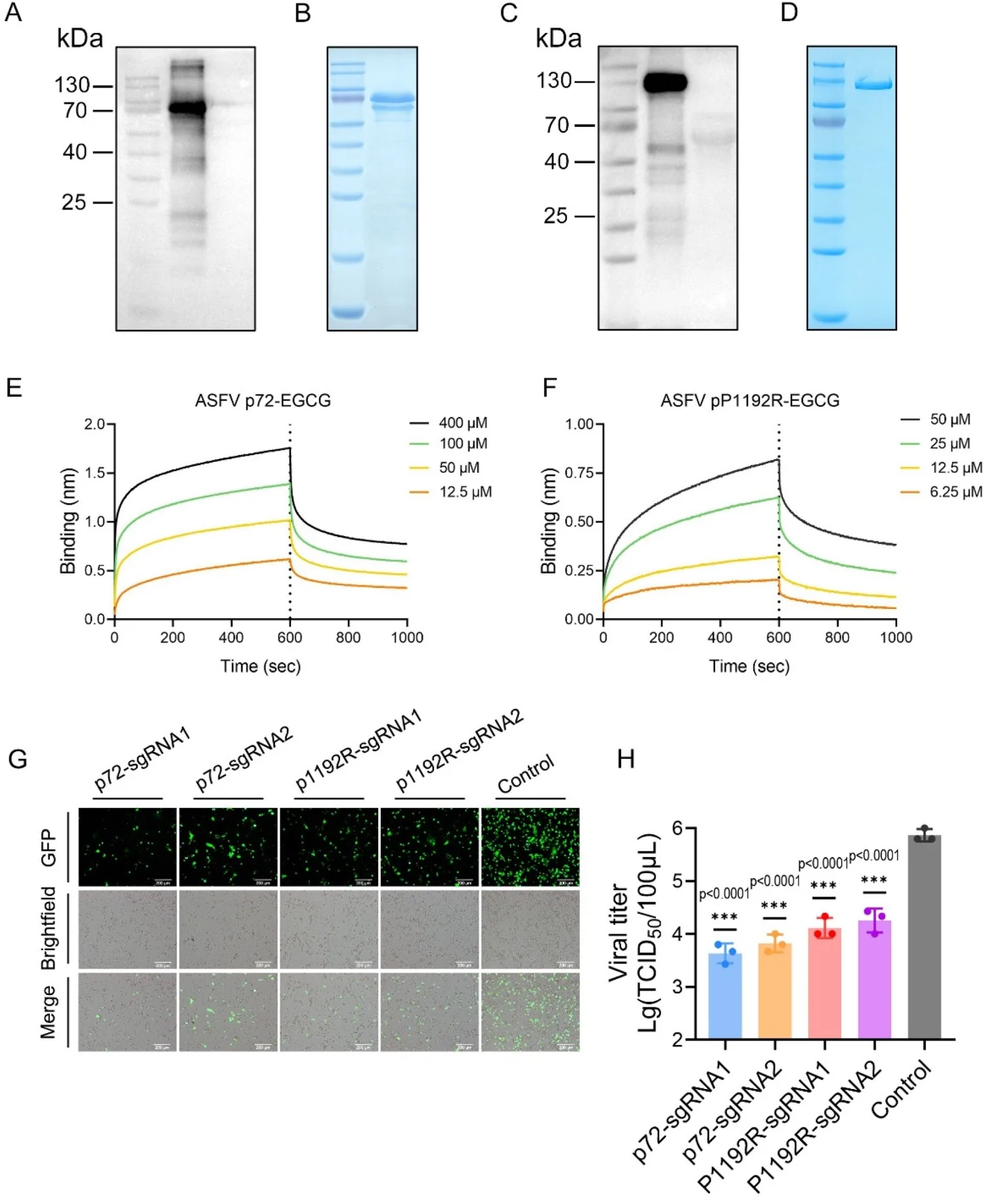
**EGCG interacts with ASFV p72 and p1192R proteins to impair viral replication.** (A, C) Western blotting analysis confirming the expression of the recombinant p72 (A) and p1192R (C) proteins. (B, D) SDS-PAGE analysis followed by Coomassie blue staining of the purified recombinant p72 (B) and p1192R (D) proteins. (E, F) BLI analysis showing the real-time binding kinetics of EGCG to immobilized p72 (E) and p1192R (F) at the indicated compound concentrations. (G, H) Effects of p72 and P1192R gene ablation on ASFV replication. LLC-PK1-Cas9 cells stably expressing specific sgRNAs (p72-sgRNA1, p72-sgRNA2, P1192R-sgRNA1, P1192R-sgRNA2) or a non-targeting control sgRNA, established by lentiviral transduction followed by puromycin selection. Cells were infected with ASFV-GFP at an MOI of 0.5. Viral replication was visualized by fluorescence microscopy (G), and progeny viral titres were quantified by TCID_50_ assay (H). Scale bars, 200 μm in all microscopy images. Data are presented as the mean ± SD of three independent biological replicates (n = 3), with individual data points superimposed on the bar graphs. Statistical significance was determined using One-way ANOVA followed by Dunnett’s multiple comparison test against the control group. ns, not significant; *p < 0.05, **p < 0.01, ***p < 0.001.

To further validate the functional importance of p72 and P1192R in ASFV replication as well as their direct interactions with EGCG, recombinant p72 and p1192R proteins were expressed and purified. Successful protein expression and high purity were validated via Western blotting (Figure 4(A and C)) and Coomassie-stained SDS-PAGE (Figure 4(B and D)), respectively. Crucially, the native folding and antigenicity of both recombinant proteins were verified by robust specific binding to serum from ASFV-positive convalescent pigs (Supplementary Figures S2B and S2C), confirming suitable native conformations for subsequent binding assays.

BLI was then applied to characterize the direct binding between EGCG and the two viral proteins. Both p72 (Figure 4(E)) and p1192R (Figure 4(F)) exhibited strong, dose-dependent binding to EGCG, with dissociation constants (KD) of 5.42×10^−8^ M (p72-EGCG) and 3.14×10^−7^ M (p1192R-EGCG), respectively, suggesting that EGCG restricts ASFV replication through direct targeting of these indispensable viral proteins. Furthermore, to ensure the specificity of the CRISPR/Cas9 editing and rule out non-specific global inhibition of viral replication, the specific targeted disruption of the *p72* and *P1192R* genes was validated. Consistent with established foundational studies demonstrating that p72 acts as the indispensable major capsid protein for virion assembly [49] and P1192R serves as the essential topoisomerase for genome unlinking [50,51], sgRNA-mediated gene ablation confirmed that the p72 and P1192R genes are essential for efficient ASFV replication (Figure 4(G)). Moreover, viral titres were significantly decreased following sgRNA-mediated editing of p72 and P1192R, with a particularly pronounced 2-log_10_ reduction observed for the p72 gene (Figure 4(H)). Together with the BLI binding data, these results demonstrate that the direct interaction of EGCG with p72 and p1192R fundamentally contributes to its anti-ASFV activity. Collectively, this study provides multi-dimensional evidence supporting the specific targeting mechanism of EGCG toward ASFV p72 and p1192R, laying a theoretical foundation for the development of EGCG-based anti-ASFV strategies.

### EGCG alleviates lipid metabolism dysregulation induced by ASFV infection

**Figure 5.**
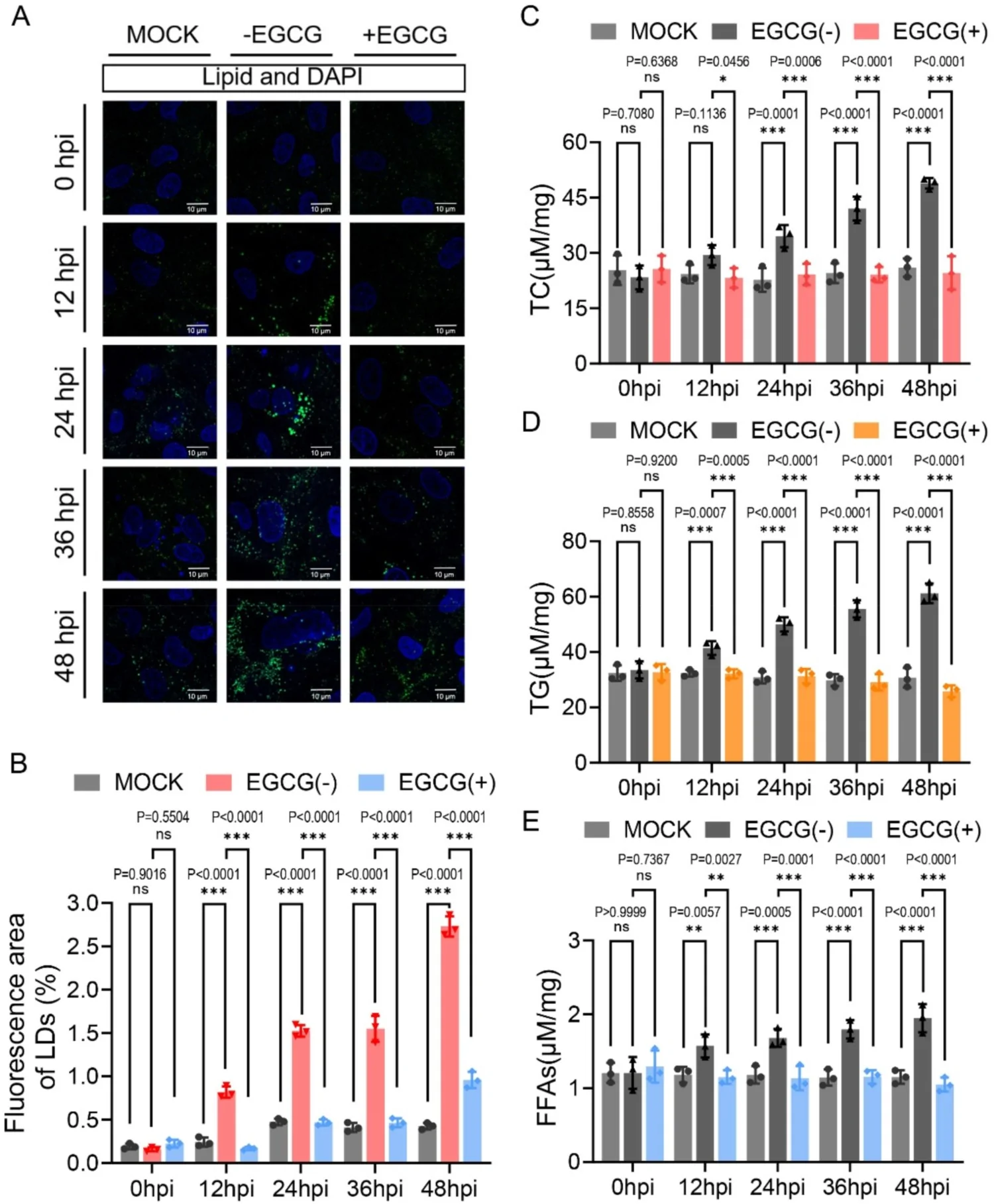
**EGCG attenuates ASFV-induced lipid droplet accumulation and cellular lipid content elevation.** (A) Representative fluorescent micrographs of lipid droplets (green, BODIPY staining) and nuclei (blue, DAPI staining) in LLC-PK1 cells under mock, ASFV-infected (-EGCG), and ASFV-infected (+EGCG) conditions at 0, 12, 24, 36, and 48 hpi. Cells were infected with ASFV at an MOI of 0.5 and treated with EGCG (30 μM). Scale bars, 10 μm. (B) Quantification of the lipid droplet fluorescence area in (A), normalized to the total cellular area of each microscopic field. Fluorescence quantification was performed using ImageJ software. (C–E) Analysis of cellular lipid contents in LLC-PK1 cells treated as in (A). (C) Total cholesterol (TC), (D) triglycerides (TG), and (E) free fatty acids (FFAs) were extracted and measured using commercial assay kits at the indicated time points. Data in (B–E) are presented as the mean ± SD of three independent biological replicates (n = 3), with individual data points overlaid on each bar graph. Statistical significance was determined using Two-way ANOVA followed by Bonferroni's multiple comparison test to compare the ASFV-infected (-EGCG) group with the mock group, and the ASFV-infected (+EGCG) group with the ASFV-infected (-EGCG) group at each time point. ns, not significant; *p < 0.05, **p < 0.01, ***p < 0.001.

Previous studies have reported that lipid metabolism is critical for ASFV replication and that EGCG exhibits regulatory effects on cellular lipid metabolism [36]. Accordingly, we further investigated whether EGCG inhibits ASFV replication by regulating lipid metabolism pathways. Cells were infected with ASFV in the presence or absence of EGCG, and intracellular lipid droplet formation was examined by BODIPY staining. The results showed that ASFV infection markedly increased the number of intracellular lipid droplets, whereas EGCG treatment significantly attenuated lipid droplet accumulation induced by viral infection (Figure 5(A and B)). Subsequently, cellular lipid contents were measured in EGCG-treated cells at 0–48 h post-ASFV infection. Consistent with the imaging data, the results showed that ASFV infection significantly increased the intracellular contents of total cholesterol (TC) (Figure 5(C)), triglycerides (TG) (Figure 5(D)), and free fatty acids (FFAs) (Figure 5(E)), whereas EGCG treatment effectively suppressed the accumulation of these lipids, suggesting that EGCG may act by inhibiting lipid synthesis. Collectively, these results confirm that EGCG impedes ASFV-induced lipid droplet formation and lipid accumulation, thereby impairing the intracellular lipid environment required for efficient viral replication.

### EGCG inhibits ASFV replication by regulating lipid synthesis through the AMPK pathway

**Figure 6.**
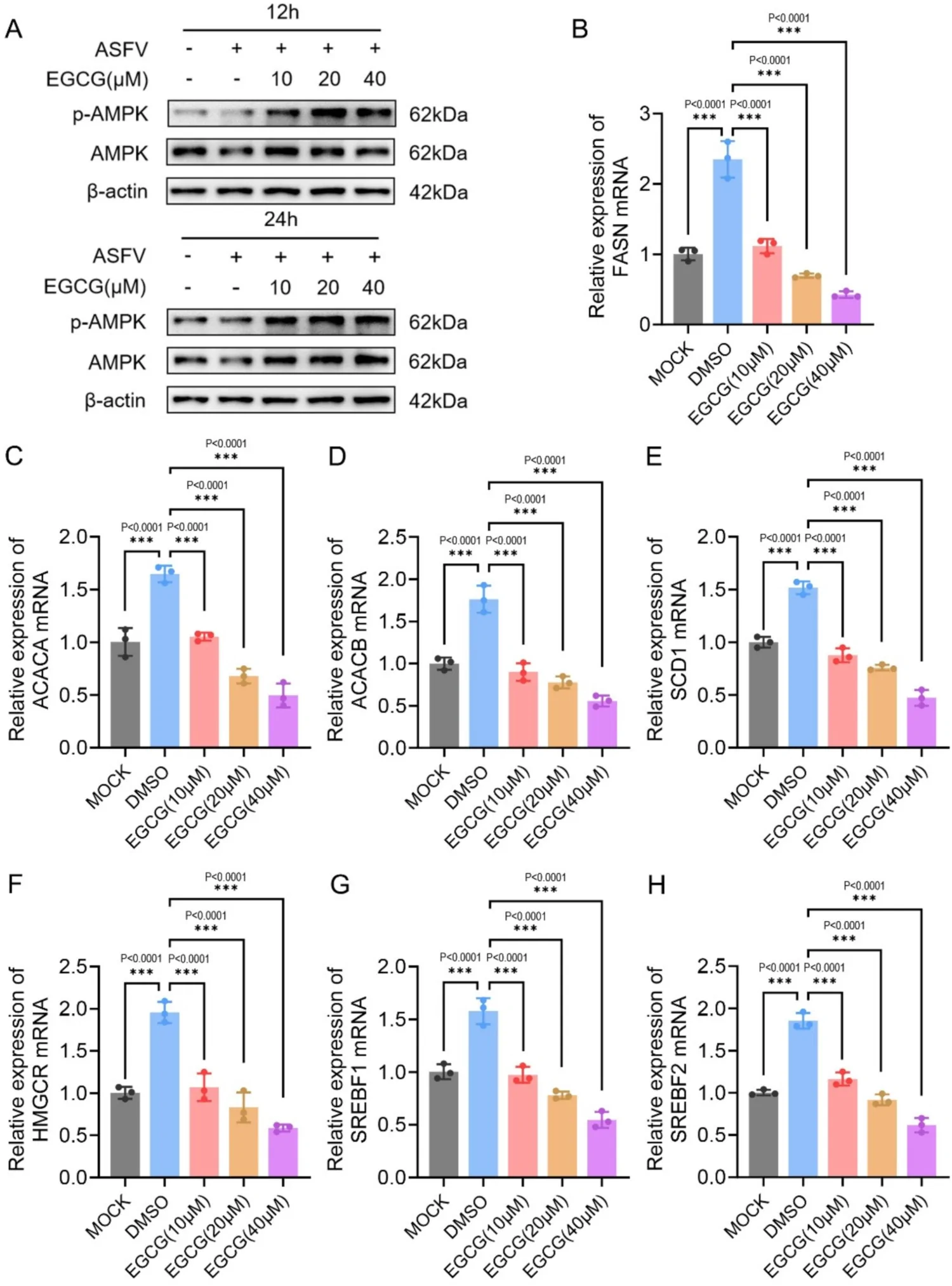
**EGCG inhibits ASFV-induced lipid accumulation by regulating the AMPK signalling pathway and lipogenic gene expression.** (A) Western blotting analysis of phosphorylated AMPK (p-AMPK) and total AMPK protein levels in LLC-PK1 cells. Cells were infected with ASFV (MOI = 1) for 2 h, followed by EGCG treatment, and harvested at 12 and 24 hpi. (B–H) Relative mRNA expression levels of key lipogenic genes in LLC-PK1 cells treated as described in (A), determined by qRT-PCR at 24 hpi. (B) *FASN*, (C) *ACACA*, (D) *ACACB*, (E) *SCD1*, (F) *HMGCR*, (G) *SREBF1*, (H) *SREBF2*. Data in (B–H) are presented as the mean ± SD of three independent biological replicates (n = 3), with individual data points overlaid on each bar graph. Statistical significance was determined using One-way ANOVA followed by Dunnett’s multiple comparison test to compare the Mock group and the EGCG-treated groups against the ASFV-infected DMSO control group. ns, not significant; *p < 0.05, **p < 0.01, ***p < 0.001.

The AMPK signalling pathway is a core physiological energy sensor that maintains cellular and systemic energy homeostasis [52,53]. Accumulating evidence has demonstrated that numerous pharmacological agents, natural compounds, and metabolic regulators exert biological functions via modulating AMPK activity. Here, we further investigated whether EGCG suppresses ASFV replication through AMPK pathway activation. The results showed that EGCG activated the AMPK signalling pathway (Figure 6(A)) in a dose-dependent manner by elevating AMPK phosphorylation (p-AMPK) in ASFV-infected cells, with sustained p-AMPK upregulation observed at 12 and 24 h post-treatment. To ensure the physiological relevance of this mechanistic pathway, we further validated this phenotype in primary host cells. Consistent with the cell line data, immunoblotting analysis confirmed that EGCG similarly induces robust AMPK signalling activation in ASFV-infected PAMs (Supplementary Figure S7).

As AMPK plays a central role in regulating lipid metabolism and EGCG has been reported to reduce cellular lipid accumulation [45], we investigated the regulatory effects of EGCG on lipid metabolism during ASFV infection. The results revealed that ASFV infection upregulated the expression of multiple lipid synthesis-related genes, including acetyl-CoA carboxylase alpha (*ACACA*) (Figure 6(C)), acetyl-CoA carboxylase beta (*ACACB*) (Figure 6(D)), fatty acid synthase (*FASN*) (Figure 6(B)), 3-hydroxy-3-methylglutaryl-CoA reductase (*HMGCR*) (Figure 6(F)), stearoyl-CoA desaturase 1 (*SCD1*) (Figure 6(E)), sterol regulatory element-binding transcription factor 1 (*SREBF1*) (Figure 6(G)), and sterol regulatory element-binding transcription factor 2 (*SREBF2*) (Figure 6(H)). EGCG treatment significantly downregulated these genes in a dose-dependent manner and persistently suppressed their expression in ASFV-infected cells. To functionally verify whether AMPK activation mediates the EGCG-induced restriction of ASFV replication, we performed a rescue assay using the AMPK inhibitor dorsomorphin. After establishing safe working concentrations in LLC-PK1 and PAM cells (Supplementary Figure S5), we found that while dorsomorphin alone did not significantly alter viral titres (Supplementary Figures S6A and S6B), co-treatment partially restored the ASFV-GFP replication suppressed by EGCG (Supplementary Figures S6C and S6D). Collectively, these results demonstrate that EGCG inhibits ASFV replication by activating the AMPK pathway to downregulate the expression of host lipid synthesis-related genes.

### EGCG exhibits broad-spectrum antiviral activity against multiple porcine viruses

**Figure 7.**
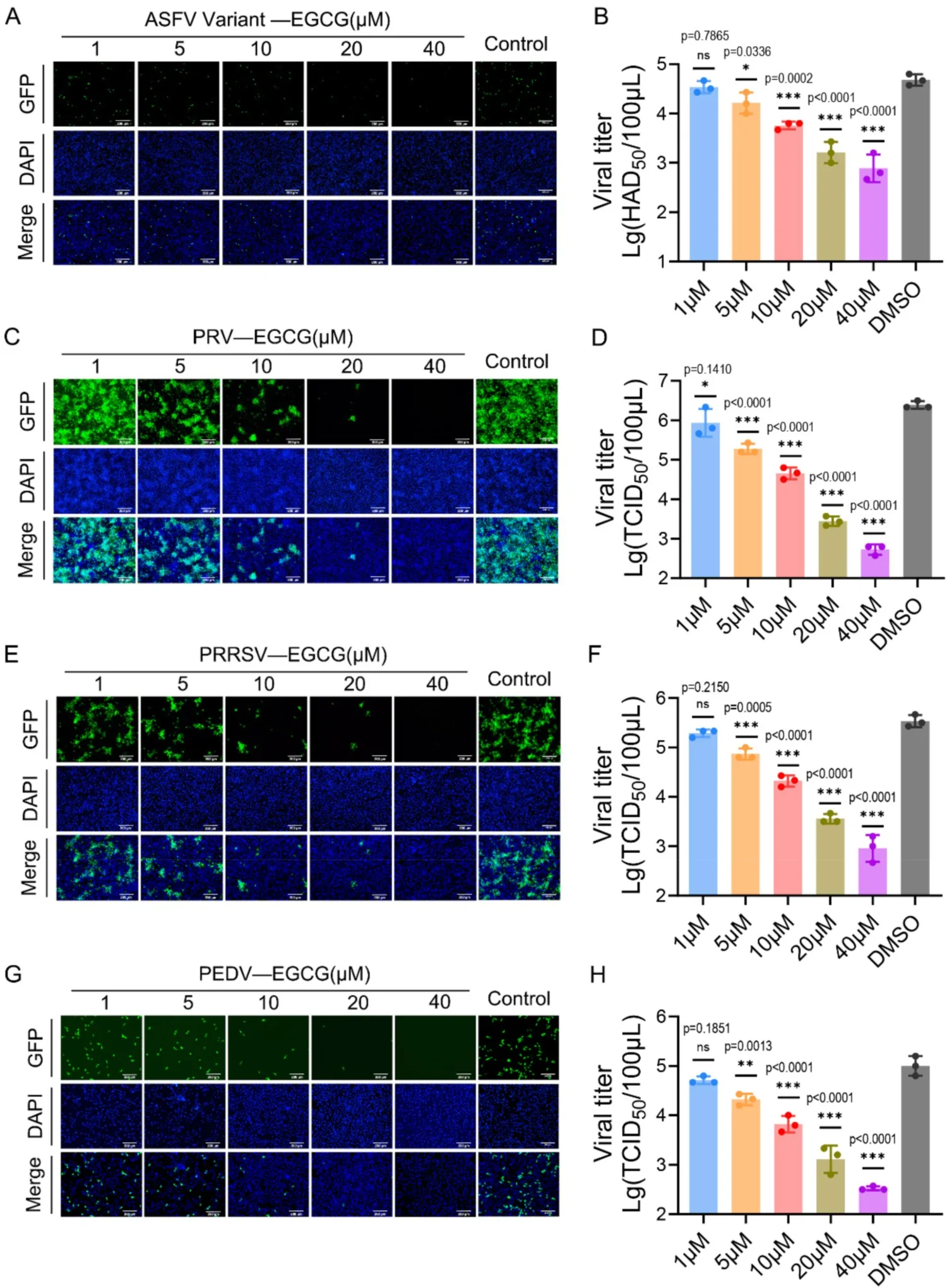
**EGCG exhibits broad-spectrum antiviral activity against multiple porcine viruses.** (A, B) Inhibitory effects of gradient EGCG on recombinant genotype I and genotype II ASFV variant (ASFV-Var) replication. PAM cells were infected with ASFV variant at an MOI of 0.5 and treated with EGCG for 48 h. Viral replication was visualized by fluorescence microscopy (A), and viral titres were quantified by HAD₅₀ assay (B). (C, D) Inhibition of PRV replication by EGCG. PK-15 cells were infected with PRV-GFP at an MOI of 0.1 and treated with EGCG for 36 h. Viral replication was visualized by fluorescence microscopy (C), and viral titres were quantified by TCID₅₀ assay (D). (E, F) Inhibition of PRRSV replication by EGCG. Marc-145 cells were infected with PRRSV-GFP at an MOI of 0.1 and treated with EGCG for 48 h. Viral replication was visualized by fluorescence microscopy (E), and viral titres were quantified by TCID₅₀ assay (F). (G, H) Inhibition of PEDV replication by EGCG. Vero cells were infected with PEDV-GFP at an MOI of 0.1 and treated with EGCG for 36 h. Viral replication was visualized by fluorescence microscopy (G), and viral titres were quantified by TCID₅₀ assay (H). Scale bars, 200 µm. Data are presented as the mean ± SD of three independent biological replicates (n = 3), with individual data points overlaid on each bar graph. Statistical significance was determined using One-way ANOVA followed by Dunnett’s multiple comparison test against the DMSO control group. ns, not significant; *p < 0.05, **p < 0.01, ***p < 0.001.

To explore the potential application of EGCG against porcine viral diseases, we further evaluated its inhibitory effects on pseudorabies virus (PRV), porcine reproductive and respiratory syndrome virus (PRRSV), porcine epidemic diarrhea virus (PEDV), and a recombinant genotype I and genotype II ASFV (ASFV-Var) [54]. Viral replication was assessed by observing fluorescence signals in cells infected with PRV (Figure 7(C)), PRRSV (Figure 7(E)), PEDV (Figure 7(G)), and ASFV-Var (Figure 7(A)) following treatment with different concentrations of EGCG. The results showed that fluorescence signals in virus-infected cells gradually diminished with rising EGCG concentrations and were nearly abolished at 40 μM, indicating that EGCG significantly suppresses the replication of all four viruses. Viral titration further revealed a concentration-dependent reduction in viral titres compared with the control group. For PRV, the viral titre was approximately 10^6.4^ TCID₅₀ / 100 μL in the control group but decreased to around 10^2.7^ TCID₅₀ / 100 μL following 40 μM EGCG treatment (Figure 7(D)). PRRSV (Figure 7(F)), PEDV (Figure 7(H)), and ASFV-Var (Figure 7(B)) displayed similar inhibitory patterns. These results confirmed that EGCG exerts profound broad-spectrum antiviral activity against PRV, PRRSV, PEDV, and ASFV, providing a theoretical basis for the development of EGCG-based therapeutics against diverse porcine viral pathogens.

## Discussion

The global swine industry is confronting an unprecedented threat posed by ASF, as there are currently no commercially available effective vaccines or therapeutics to control this disease. This study demonstrates that EGCG exerts potent anti-ASFV activity via a dual synergistic mechanism, which mitigates the inherent limitations of single-target antiviral strategies. Specifically, this dual mechanism combines direct viral targeting and host metabolic regulation, achieving a more comprehensive antiviral effect compared to single-target approaches. On one hand, EGCG directly binds to p1192R and p72, two key proteins of ASFV, as confirmed by molecular docking and BLI analysis. These binding interactions disrupt viral genome replication (mediated by P1192R) [50,51] and capsid assembly (mediated by p72) [49], both of which are critical for ASFV proliferation. At the molecular level, p72 serves as the major ASFV capsid protein and is absolutely indispensable for capsomere trimerization and progeny virion assembly. Our structural analysis of the p72-EGCG complex reveals a compelling mechanistic basis for its inhibition. The spatial occupation of EGCG at these highly conserved structural hotspots imposes a significant steric clash. Simultaneously, EGCG targets p1192R, the unique viral type II topoisomerase that is strictly required for the unlinking of newly replicated viral genomes during ASFV DNA replication. EGCG engages p1192R through an extensive hydrogen-bonding network distributed across multiple essential functional domains. This multi-domain blockade directly interferes with both the DNA cleavage and the subsequent strand transport steps of the topoisomerase catalytic cycle, thereby halting viral genome replication.

On the other hand, EGCG modulates host lipid metabolism by activating the AMPK signalling pathway, which in turn downregulates lipid synthesis-related genes and reverses ASFV-induced accumulation of lipid droplets, TC, TG, and FFAs. This dual synergistic mechanism exerts a coordinated inhibitory effect on ASFV, characterized by direct targeting of viral components and disruption of the metabolic microenvironment essential for viral replication. In contrast to single-target antiviral drugs that are prone to inducing viral resistance, the dual synergistic mechanism of EGCG reduces the risk of resistance development, as viruses are less likely to simultaneously acquire mutations that enable evasion of both viral protein targeting and host metabolic regulation. Furthermore, the identification of p1192R and p72 as direct molecular targets of EGCG provides valuable insights for the structure-based design of novel small-molecule inhibitors or targeted degradation strategies specifically targeting these key viral proteins, which would complement and enhance the therapeutic potential of EGCG in the clinical management of ASF.

Viral replication is inherently dependent on the hijacking of host cellular resources, and ASFV has evolved sophisticated mechanisms to exploit host lipid metabolism for energy supply and the synthesis of structural materials [36]. The study confirms that ASFV infection significantly upregulates the expression of lipid synthesis-related genes and induces lipid droplet accumulation, which is consistent with previous findings demonstrating that lipid droplets serve as critical reservoirs for viral envelope assembly and genome replication [36,55]. The capacity of EGCG to disrupt this metabolic hijacking process through AMPK activation underscores the significance of targeting host metabolic pathways in the development of antiviral agents. As a central “energy sensor” in cells, the AMPK pathway, upon activation by EGCG, not only inhibits de novo lipid synthesis but also promotes lipid oxidation, thereby effectively depriving ASFV of the lipid substrates essential for its life cycle progression. Notably, this regulatory effect of EGCG is not restricted to ASFV-infected cells, and accumulating evidence has shown that EGCG modulates lipid metabolism in various cell types, indicating a conserved regulatory mechanism that could be generalized to other lipid-dependent viral pathogens [45,56]. Moreover, the dose-dependent downregulation of lipid synthesis genes by EGCG further supports its potential as a tunable therapeutic agent, allowing for tunable modulation of host metabolism to counter viral infection. Our findings closely align with a recent study demonstrating that theaflavin, another natural tea-derived polyphenol, also inhibits ASFV replication by activating the AMPK pathway to disrupt lipid metabolism [56]. While both theaflavin and EGCG underscore the profound vulnerability of ASFV to host metabolic reprogramming via AMPK activation, our study reveals that EGCG possesses an additional, direct antiviral dimension. This unique dual-synergistic mechanism offers a more comprehensive antiviral coverage and inherently establishes a higher genetic barrier to viral resistance.

Importantly, this raises a critical virological question: whether the observed reduction in lipid accumulation represents a mechanism contributing to viral inhibition or a consequence of reduced viral replication. We propose that it is a highly synergistic combination of both phenomena. As an intrinsic host metabolic modulator, EGCG proactively activates AMPK to create a lipid-depleted state hostile to viral replication. Simultaneously, because EGCG structurally cripples the virus by directly binding the key viral proteins p72 and p1192R, the effectively aborted viral replication means the virus can no longer actively hijack host metabolic fluxes. This bidirectional interpretation significantly elevates the logical depth of our proposed dual-target model.

In addition to its anti-ASFV activity, this study further demonstrated that EGCG has an inhibitory effect on the replication of other economically significant porcine viral pathogens, including PRV, PRRSV, and PEDV. This broad-spectrum antiviral activity is presumably attributed to the capacity of EGCG to target evolutionarily conserved host cellular pathways, such as lipid metabolism, that are critical for the replication of a diverse range of viral species [30]. For instance, like ASFV, PRV and PRRSV also exhibit a reliance on host lipid metabolism for viral envelope biosynthesis and viral particle assembly, rendering them susceptible to the metabolic regulatory effects mediated by EGCG [57,58]. Additionally, the anti-inflammatory and antioxidant properties of EGCG may further contribute to its broad-spectrum antiviral efficacy by mitigating virus-induced dysregulation of host immune responses, which otherwise exacerbate tissue damage and facilitate viral dissemination [30]. The development of a single agent with activity against multiple porcine viruses could help address a critical gap in current swine disease control strategies, which rely on the concurrent use of multiple vaccines and antiviral drugs. The natural origin, low cytotoxicity profile, and broad-spectrum antiviral activity of EGCG position it as a promising candidate for the development of multi-target antiviral preparations, potentially reducing the economic burden of porcine viral diseases on the global swine industry.

Compared to conventional synthetic antiviral agents, EGCG possesses several distinct advantages. As a natural compound derived from green tea, EGCG has a well-characterized safety profile in both humans and animals, thereby minimizing the risk of adverse effects associated with synthetic drugs [29]. Its dual mechanism of action, as discussed earlier, provides a unique strategy to combat ASFV and reduce the likelihood of resistance development. Furthermore, the results demonstrate that EGCG effectively inhibits ASFV replication across multiple cell types and against the prevalent ASFV variant, indicating its potential applicability to diverse ASFV genotypes and clinical scenarios. Despite these promising attributes, several critical limitations must be addressed prior to the translational application of EGCG into clinical practice for ASF control. Currently, the anti-ASFV efficacy of EGCG has been evaluated exclusively *in vitro*. Due to stringent national biosafety regulations and constraints regarding access to high-containment animal facilities (ABSL-3) required for highly pathogenic ASFV challenge studies, *in vivo* validation could not be conducted at this stage. Future studies utilizing swine or appropriate surrogate infection models are critically essential. Translating these findings into therapeutics will require comprehensive *in vivo* assessments, such as evaluating pharmacokinetics and bioavailability, monitoring viral loads in target organs, and determining pathological lesion scores. Furthermore, the inherent stability and aqueous solubility of EGCG may constrain its therapeutic potential. The development of EGCG derivatives or formulations, such as nanoparticles and liposomes, with improved bioavailability and targeted delivery could enhance its antiviral activity *in vivo*. In addition, the potential synergistic effects of EGCG in combination with other antiviral agents remain unexplored, and combining EGCG with other compounds may further improve therapeutic efficacy.

In conclusion, despite these *in vivo* limitations, this study systematically demonstrates the dual mechanism by which EGCG inhibits ASFV replication, highlights its broad-spectrum antiviral potential, and underscores its advantages as a natural, safe, and multi-target antiviral candidate. These findings provide profound new insights into the design of novel anti-ASFV strategies and pave the way for the development of EGCG-based therapeutics for controlling ASF and other porcine viral diseases.

## Supplementary Material

Supplementary_Materials.docx
