# Supplementary material for: Epigallocatechin gallate (EGCG) inhibits African swine fever virus replication by targeting key viral proteins and regulating host lipid metabolism
Source: Emerg Microbes Infect. 2026 Sep 7;15(1):2731508. doi: 10.1080/22221751.2026.2731508 (PMC13591956; doi:10.1080/22221751.2026.2731508)
Supplement: Supplementary file 1 [file TEMI_A_2731508_SM5007.docx]

**Supplementary Materials**

**Title: Epigallocatechin Gallate (EGCG) Inhibits African Swine Fever Virus Replication by Targeting Key Viral Proteins and Regulating Host Lipid Metabolism**

Authors: Xinglin He, Hong Li, Ruiqi Yang, Pengfei Li, Guiqian Wang, Hua Cao, Yumei Sun, Mengjia Zhang, Yongtao Li, Kaizhi Shi, Anan Jongkaewwattana, Shengnan Ruan, Wentao Li

**Supplementary Figure S1.** **Effects of EGCG on transcription of ASFV early and late genes.** (A, B) Relative mRNA expression profiles of ASFV early gene *p30* (A) and late gene *p72* (B). Cells were infected with ASFV at an MOI of 1 and treated with EGCG (30 μM) or vehicle control. Cells were harvested at the indicated time points (0 to 24 hpi), and viral mRNA levels were quantified by RT-qPCR. Data are presented as the mean ± SD of three independent biological replicates (n = 3), with individual data points superimposed on the bar graphs. Statistical significance was determined using Two-Way ANOVA followed by Bonferroni’s multiple comparison test to compare the EGCG-treated group with the vehicle control at each time point. ns, not significant; *p < 0.05, **p < 0.01, ***p < 0.001.

**Supplementary Figure S2. Expression and serological identification of recombinant ASFV proteins.** Western blot was used to assess the expression and antigenic reactivity of recombinant ASFV proteins. (A) Immunoblotting with anti-FLAG monoclonal antibody confirmed the expression of recombinant B602L protein. (B, C) Purified recombinant p72 (B) and p1192R (C) proteins were specifically recognized by serum samples from ASFV-positive pigs. Protein molecular weight markers (kDa) are indicated on the left side of each immunoblot.

**Supplementary Figure S3. Extensive sequence conservation of the ASFV major capsid protein p72 among globally distributed isolates.** Multiple sequence alignment of the p72 protein (encoded by the *B646L* gene) from 15 representative ASFV strains, covering China/LN/2018/1, Georgia 2007/1, Benin 97/1, and BA71 reference isolates. The sequence alignment was generated and visualized to highlight conserved regions. Identical amino acid residues are shaded in solid red, demonstrating the extreme genetic stability of the p72 protein among different ASFV genotypes and geographical isolates.

**Supplementary Figure S4. Conservation analysis of ASFV DNA topoisomerase II (p1192R) sequences across distinct viral strains.** Multiple sequence alignment of p1192R protein sequences from 15 geographically and genotypically distinct ASFV isolates. Fully conserved amino acid residues are shaded dark red, demonstrating robust genetic conservation of p1192R among divergent ASFV isolates. This high inter-strain sequence conservation supports p1192R as a genetically stable target for broad-spectrum anti-ASFV therapeutics.

**Supplementary Figure S5. Cytotoxic profiling of the AMPK inhibitor dorsomorphin in LLC-PK1** **cells and primary porcine alveolar macrophages (PAMs).** (A, B) Cell viability detection in LLC-PK1 (A) and PAMs (B) following dorsomorphin treatment. Cells were incubated with graded concentrations of dorsomorphin (0–40 μM for LLC-PK1, 0–20 μM for PAMs) for 48 h; DMSO was used as vehicle control. Cellular viability was quantified using the CCK-8 colorimetric assay. All data represent mean ± SD across six independent biological replicates (n = 6), and individual data points are overlaid on bars.

**Supplementary Figure S6. AMPK pharmacological inhibition partially restores ASFV replication suppressed by EGCG.** (A) Fluorescence microscopy analysis of ASFV-GFP replication in LLC-PK1 cells. Cells were infected with ASFV-GFP (MOI = 0.5) and treated with the indicated concentrations of the AMPK inhibitor Dorsomorphin (2.5, 5, 10 μM) or DMSO vehicle control. GFP fluorescence indicates viral replication, and nuclei were counterstained with DAPI. Scale bars, 200 μm. (B) Viral titers in the supernatants of cells treated as in (A), determined by TCID_50_ assay. (C) Fluorescence microscopy analysis evaluating the rescue effect of Dorsomorphin. ASFV-GFP-infected LLC-PK1 cells (MOI = 1) were treated with EGCG (30 μM) alone, co-treated with EGCG and Dorsomorphin (2.5 or 5 μM), or treated with DMSO (Control) for 24 h. Scale bars, 200 μm. (D) Quantification of the percentage of GFP^+^ cells in panel (C). Data are presented as the mean ± SD of three independent biological replicates (n = 3), with individual data points superimposed on the bar graphs. Statistical significance was determined using One-Way ANOVA followed by Dunnett’s multiple comparison test. In (B), the Dorsomorphin-treated groups were compared against the DMSO control. In (D), the co-treatment groups and the vehicle control were compared against the EGCG-only group to evaluate the rescue efficiency. ns, not significant; *p < 0.05, **p < 0.01, ***p < 0.001.

**Supplementary Figure S7. EGCG induces AMPK signaling activation in ASFV-infected PAMs.** Immunoblotting analysis of phosphorylated AMPK (p-AMPK) and total AMPK protein abundance in PAMs. Cells were infected with ASFV (MOI = 1) for 2 h, treated with the indicated concentrations of EGCG (5, 10, 20 μM) or DMSO vehicle control, and harvested at 24 hpi for protein extraction.

**Supplementary Table S1.**

**Pharmacological profiles of EGCG against ASFV in vitro.**

| **Cell Line** | **CC50 (μM)** | **IC50 (μM)** | **Selectivity Index (SI)** | |
| --- | --- | --- | --- | --- |
| Vero | 225.1 | 6.8 | | 33.1 |
| LLC-PK1 | 180.7 | 8.3 | | 21.8 |
| PAMs | 61.03 | 5.2 | | 11.7 |

**Supplementary Table S2.**

**Molecular docking virtual screening of EGCG against ASFV essential proteins.**

| **Target ASFV Protein** | **PDB Accession** | **Binding Energy (kcal/mol)** |
| --- | --- | --- |
| **p1192R** | **8J9Z** | **-10.0** |
| **p72 (B646L)** | **6KU9** | **-9.3** |
| pA137R | 7VIV | -8.4 |
| pA151R | 7CMA | -8.1 |
| pS273R | 6LJB | -8 |
| p15 | 7BQ9 | -7.7 |
| p35 | 7BQA | -7.7 |
| p150 | 8IGL | -7.5 |
| I73R | 7VWV | -7.5 |
| pE301R | 8ITE | -7.5 |
| CP312R | 7YEQ | -7.4 |
| Pol X | 1JAJ | -7.3 |
| pNP868R | 7D8U | -7.3 |
| d318L | 8HDL | -7.3 |
| AE79L | 5UA4 | -6.7 |
| dUTPase | 6KY8 | -6.7 |
| pI215L | 7WLH | -6.7 |
| pA104R | 6LMH | -6.5 |

Table Note: Molecular docking was performed using AutoDock Vina. A focused library comprising the available 3D crystal structures of 18 essential ASFV proteins involved in viral replication, transcription, and assembly was screened against the EGCG ligand. The top two candidates, p1192R and p72, which exhibited the lowest binding free energies (indicating the strongest affinities), were selected for subsequent biochemical and functional validations in this study.

**Supplementary Table S3.**

**Summary of key interacting residues and structural domains involved in EGCG binding to ASFV p72 and p1192R.**

| **Target Protein** | **PDB ID** | **Key Interacting Residues** | **Interaction Type** |
| --- | --- | --- | --- |
| p72 (B646L) | 6KU9 | Pro481, Ile525 | Hydrogen bonds |
|  |  | His484, Asn369, Tyr178, Arg308 | Hydrogen bonds |
|  |  | Phe371 | Hydrophobic stacking |
| p1192R | 8J9Z | His620, Lys626 | Hydrogen bonds |
|  |  | Gly781 | Hydrogen bonds |
|  |  | Glu970, Tyr768, Lys1190 | Hydrogen bonds |

Table Note: Molecular interactions between EGCG and the targeted viral proteins were analyzed using AutoDock Vina. The specific interacting residues, types of intermolecular forces, and their corresponding functional domains are summarized. The strategic localization of these residues highlights the potential for EGCG to impose steric hindrance on p72 capsomere trimerization and p1192R topoisomerase catalytic cycle (DNA cleavage and strand transport).

**Supplementary Table S4.**

**Primer sequences used for real-time quantitative PCR (RT-qPCR) analysis.**

| **Primer** | **Sequence (5’-3’)** |
| --- | --- |
| FASN-F | CCACTCCAAGCAGGCGAACAC |
| FASN-R | CACGAAGGGAAGCAGGGTTGATG |
| ACACA-F | AATCAAAGAGGTTCCAGGCACAGTC |
| ACACA-R | CGTCAGCATGTCAGAAGGCAGAG |
| ACACB-F | TCCAGACAAGACAAGAAGCAAGAGC |
| ACACB-R | AGCCAGCACCAGAGTCCTCATC |
| SCD1-F | CACCACTACCATCACAGCACCTTC |
| SCD1-R | TCATTTCAGGGCGGATGTCTTCTTC |
| HMGCR-F | TCCAGTCCAGGTCAGGTGATGC |
| HMGCR-R | ACAGCAGCAGGTTTCTTGTCAGTAC |
| SREBF1-F | TGCTGGCGGAGGTCTATGTGG |
| SREBF1-R | CAGGAAGAAGCGGGTCAGAAAGTG |
| SREBF2-F | CTGGTACGCTGGCTGCTCAAG |
| SREBF2-R | CGCTGCTCTTGGCTTGATCCTC |
| GAPDH-F | CCTTCCGTGTCCCTACTGCCAAC |
| GAPDH-R | GACGCCTGCTTCACCACCTTCT |
